# Health, wealth, and medical expenditures among the elderly in rural Tanzania: experiences from Nzega and Igunga districts

**DOI:** 10.1186/s12913-023-09943-1

**Published:** 2023-09-29

**Authors:** Malale M. Tungu, Phares G. Mujinja, Paul J. Amani, Mughwira A. Mwangu, Angwara D. Kiwara, Lars Lindholm

**Affiliations:** 1 Department of Development Studies, School of Public Health and Social Sciences, Muhimbili University of Health and Allied Sciences, Dar Es Salaam, Tanzania; 2 Department of Health Systems Management, School of Public Administration and Management, Mzumbe University, Morogoro, Tanzania; 3 Epidemiology and Global Health, Umeå International School of Public Health, Umeå University, Umeå, Sweden

**Keywords:** Health expenditure, Wealth index, QoL, EQ-5D, Elderly, Tanzania

## Abstract

**Background:**

The per capita health expenditure (HE) and share of gross domestic product (GDP) spending on elderly healthcare are expected to increase. The gap between health needs and available resources for elderly healthcare is widening in many developing countries, like Tanzania, leaving the elderly in poor health. These conditions lead to catastrophic HEs for the elderly. This study aimed to analyse the association between measures of health, wealth, and medical expenditure in rural residents aged 60 years and above in Tanzania.

**Methods:**

The data of this study were collected through a cross-sectional household survey to residents aged 60 years and above living in Nzega and Igunga districts using a standardised World Health Organization (WHO) Study on Global Ageing and Adult Health (SAGE) and European Quality of Life Five Dimension (EQ-5D) questionnaires. The quality of life (QoL) was estimated using EQ-5D weights. The wealth index was generated from principal component analysis (PCA). The linear regression analyses (outpatient/inpatient) were performed to analyse the association between measures of health, wealth, medical expenditure, and socio-demographic variables.

**Results:**

This study found a negative and statistically significant association between QoL and HE, whereby HE increases with the decrease of QoL. We could not find any significant relationship between HE and social gradients. In addition, age influences HE such that as age increases, the HE for both outpatient and inpatient care also increases.

**Conclusion:**

The health system in these districts allocate resources mainly according to needs, and social position is not important. We thus conclude that the elderly of lower socio-economic status (SES) was subjected to similar health expenditure as those of higher socio-economic status. Health, not wealth, determines the use of medical expenditures.

## Background

Aging and increasing health expenditure (HE) for the elderly are two major challenges likely to influence the global health systems. This results in a sharp increase in the per capita HE and shares of gross domestic product (GDP) of many countries on healthcare [1–3]. To accommodate the healthcare needs of the elderly, governments are challenged to put in place strategies that will improve the health service infrastructure for providing services, especially to the rural population [4]. In many low- and middle-income countries (LMICs), Tanzania inclusive, there is an increasing gap between health needs and the available resources for elderly healthcare [5]. Insufficient resources have made it difficult to fund and implement the needed healthcare services. Thus, priority setting is considered an important process that will enable countries to decide how best limited health resources should be allocated among competing programmes. This process occurs at all levels of healthcare systems and is one of the most important issues in healthcare management today [6].

Welfarism is the school of thought that believes the goodness of a state of affairs depends on the set of individual utilities [7]. This concept helps decision-makers during the priority setting process to focus that resource allocation should depend on the utility obtained by individual people. For the extra-welfarism during the priority setting process, the decision-maker believes that resource allocation should depend on factors other than a utility like health [8]. The main idea in extra-welfarism about priority setting is to maximise population health. The best strategy to achieve this goal is certainly to allocate healthcare resources according to need, similar to views in medicine and public health [9]. Importantly, extra welfarism thus rejects individual demand, i.e. ability to pay, as the mechanism to allocate resources in healthcare [8, 10]. For the developed countries, except the US, medical care is available to all its people, regardless of income, age, or residence [5]. However, things are different in many LMICs. For example, in both Tanzania and Nigeria, there are no functional social security mechanisms for elderly people, and households with elderly people are responsible for (geriatric) healthcare costs. This may increase the risk for catastrophic HEs, especially for poor households [11], or people will not use healthcare even when strongly needed.

Most of the elderly in rural areas are poor (many live on subsistence) and cannot afford escalating healthcare costs [4, 12, 13]. Therefore, elderly people are likely to be the victims of catastrophic HEs each time they are admitted to inpatient care. Catastrophic HE is defined as out-of-pocket (OOP) spending for healthcare services that exceeds 40% of the household’s income [14–16].

In Tanzania, catastrophic HE among the elderly is influenced by different financing mechanisms, including cost-sharing in the healthcare services that were introduced in the early 1990s in the country. Cost-sharing becomes a burden to vulnerable groups including the elderly because of their low incomes. Although the government has put in place policies with a focus on enhancing free access to health services to the elderly population, most of the elderly still incur costs associated with medicines, diagnostic tests, and other related services upon their utilisation of healthcare services [4, 17]. However, to qualify for an exemption, a verification process must be fulfilled before exemption identity is granted. As it is now, the provision of free services to the elderly has become another challenge to the healthcare system because there are no clear guidelines that guide the health workers at the facility and the funds that subsidise the process, i.e. the compensation of the exemption at the health facility [18]. This makes effective and efficient implementation of this policy difficult [19].

The healthcare models from most developed countries can be applied by implementing policies based on taxes or public health insurance (risk pooling), which will make healthcare services free or almost free. This means that those with the highest needs should not be restricted by the cost to access healthcare services.

The proxy for healthcare needs can be quality-adjusted-life-years (QALYs) weights. Most of the elderly suffer from one or several chronic diseases. This makes the elderly have low QALY weights and automatically have a high need for healthcare [17, 20, 21]. If the low QALY weights among the elderly are associated with a high cost for healthcare and vice versa, then there should be a fair allocation. This means that those with the highest health needs (low QALY weights) should get more health resources than those with low health needs (high QALY weights). The use of healthcare services should be determined by health needs rather than by people’s ability to pay (social position), which would lead to unfair allocation of resources. Decision-makers probably strive for healthcare distributed according to need, but in real life, they have problems achieving such a model because it requires that the rich and healthy pay a bigger share. Social position can be measured by socio-economic status (SES), which is a proxy of wealth among the elderly. SES incorporates assets, education, income, and occupation [22] and is calculated using principal component analysis (PCA).

This study explains the concept of whether the health care system in Tanzania is fair and that health expenditures are determined by need and not by the ability to pay. Available studies on the relationship between health, wealth and medical expenditures are scanty and not specific to the healthcare services of the elderly. Most of them focus on the health factors associated with out-of-pocket and catastrophic health expenditures [2], the impacts of ageing on the health status, quality of life and well-being [23] and the relationship between health and wealth on lifestyle among the employed people compared to the previous generation [24]. This study, therefore, sought to fill the gap by making an in-depth analysis of the association between measures of health, wealth, and medical expenditure in rural residents aged 60 years and above in Tanzania. Specifically, this study aimed to investigate whether the elderly use of healthcare in Tanzania is determined by people’s health or their wealth.

## Methods

### Setting of the study

This study is part of the larger project that was conducted in Tabora region in the central western part of Tanzania. It used primary data which seeks to understand the association between measures of health, wealth, and medical expenditure among rural residents aged 60 years and above that was collected in two districts of (Nzega and Igunga between July and September 2017. The total population of these districts is 901,979 (Nzega is 502,252 and Igunga is 399,727) of which 6% constitute elderly people. The choice of Igunga and Nzega districts was influenced by the fact that these districts are predominantly rural and most the elderly people live a rural life [25, 26]. Since Igunga district was the first district in Tanzania to implement community health funds (CHFs) in 1996, these two districts were chosen for logistical reasons, as they are neighboring one another.

### Study design, sampling techniques, and sample size

Data were collected from 1,899 elderly (1,179 in Nzega and 720 in Igunga). A multistage sampling method was used to select wards, villages and households with an elderly person with the help of ward and hamlet leaders. Firstly, we purposely selected 14 wards (seven from each district) based on the population size and proximity between the wards. Secondly, we randomly selected 58 villages that were geographically reachable using a lottery method. Thirdly, a systematic sampling technique was used to identify and select 25 to 44 households by selecting every second household with an elderly person from each village, depending on the size of the village. Lastly, one respondent from each household was selected randomly. Consideration was given to gender balance in the selection of the respondents. The required sample size was determined to be 733 participants, based on a prevalence of the outcome of catastrophic health expenditure of 40%, a design effect of 2, and considering a 95% confidence interval (CI) and 80% power [27]. We doubled the sample to have a representative group of men and women. The following is the formula used: *n* = Z^2^ [P(1-P)*D]/ε^2^, n = [1.96^2^ * 0.4(1–0.4)*2]/0.05^2^ = 733, whereby Z = Z-score, P = Prevalence of the outcome, D = Design effect, ε^2^ = Marginal effect.

### Measurement of health need

EQ-5D instrument was used to measure the health needs using five dimensions (mobility, self-care, usual activities, pain/discomfort, and anxiety/depression). The dimensions were divided into three levels (no problems, some problems, and severe problems). The position for each health state is on a scale of 0 and 1, whereby 0 represents death and 1 represents perfect health. QALY weights can be calculated by considering the preferences through direct methods like time-trade-off and visual analogue scale [28]. We converted the EQ- 5D-3L answers into a single number to have QALY weights, applying the value sets from Zimbabwe (ZW), which is close to our settings [29].

### Measurement of costs

HE includes costs per visit, bed day costs, and transport costs at the health facility as per WHO^1^ suggestions. HE also included registration fees, laboratory test costs, and medicine payment costs. The WHO categorises the costs according to the public, private, or faith-based organisation (FBO) health facility. This was used to construct an index with three types of costs: costs for those attending to the public, private, and charity or FBO health facility. These costs were added to the costs of the registration fee, medicines, lab tests, and transport costs to arrive at total costs.

### Measurement of wealth

In addition, the wealth index was created in terms of the SES index that was constructed. In constructing PCA using the Stata programme, several steps were performed. The SES index in this study comprises material ownership (assets) and occupation. The SES variable was constructed to assess the wealth index of the elderly in Nzega and Igunga by the following steps:

Step 1: Selection of variables, which were used for PCA (electricity, bicycle, motorcycle, television, radio, pressing iron, refrigerator, mobile phone, car, computer, land, cattle, income, and occupation) by using a command *tabulate var*_*i…*_*var*_*n*_. Step 2: Generate dummy variables using a command *tabulate var*_*,*_* gen(var).* Step 3: Checking for multicollinearity (correlation of the coefficient between variables) by using a command *corr* and listing all variables to be used for PCA. Step 4: Checking the assumptions for PCA (Bartlett's Test and Kaiser–Meyer–Olkin [KMO] measure of sampling adequacy), by using a command *factortest* which found the value of KMO > 0.5. Step 5: Thereafter, the PCA was constructed, and *Eigen* factors and *Eigenvalues* were checked by using a command *pca var*_*1*_*, var*_*2*_*, …var*_*n*_*, means components (3)* and then *rotate, varimax.* Step 6: We graphed the *Eigenvalue* using the command *greigen* following the *pca* command. Step 7: We computed the component score using a command *predict f1 f2 f3*. Step 8: Finally, the component scores were categorised into quintiles to be used in the regression as a predictor using the command *xtile ixpurw* = *f1, nq(5)* [30, 31]*.*

### Other variables

Demographic variables were used as predictors (age, sex, marital status, and education level). This included sex (female/males), age (categorised into 60–69, 70–79, 80–89, and 90 +), and marital status, which included those who were married (i.e., currently married and cohabiting) and non-married (i.e., widows, separated, never married, and divorced). Education included no formal education, low (4–8 years, primary education), middle (11–14 years, secondary education), and high (university education).

### Data analysis

Linear regression analyses were performed to analyze the association between measures of health, wealth, medical expenditure (outpatient/inpatient), and socio-demographic variables. The multivariable regression model using simple linear regression analysis was performed to assess the association between HE, quality of life (QoL), and SES among the elderly.

### Model specification

**Fig. 1:**
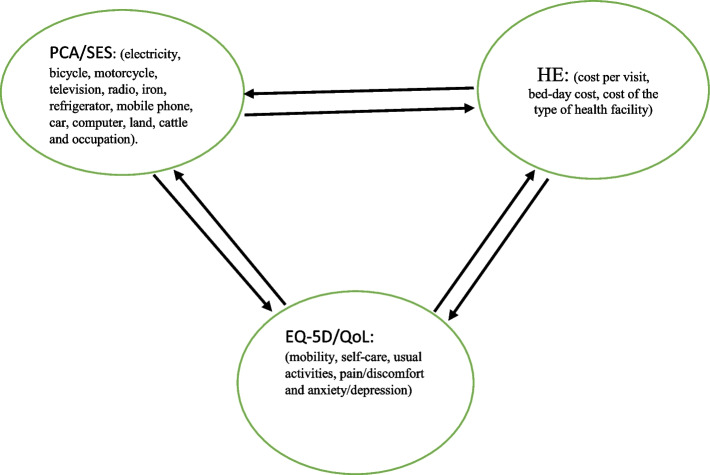
The relationship among healthcare use, QoL, and SES. Source: Author’s construction

The mutual relationship is presented in Fig. 1, which shows the relationship among HE, QoL, and SES. When the elderly are ill, they need treatment i.e. health demand, and after receiving the treatment, they may improve their health status (QoL). This means that demand for health can lead to the improvement of health of the elderly. Also, QoL and the need for healthcare (health demand) are influenced by the level of wealth/income of the individual elderly. The same applied to the vice-versa relationship since those with a high level of wealth can probably get more and/or better healthcare, which improves their health status [32]. However, our cross-sectional data do not allow us to investigate the mutual relationships. Only associations between our proxies for need (demand) and social position and the use of healthcare were analysed in the study.

## Results

### Characteristics of the respondents

Of the 1,899 respondents, 51% were women and 49% were men as indicated in Table 1. About 62% of the respondents were still engaging themselves in economic activities, one of which is subsistence agriculture Compare to male, elderly women engaged more in small business. In rural areas, most of the elderly use firewood (74.6%) as a source of energy.

**Table 1:** Summary of demographic and socio-economic characteristics of the respondents: frequency (percentages) (*N* = 1,899)

	**Female**	**Male**	**Total**
**Age group**			
60–69	594(61.3)	517(55.59)	1111(58.5)
70–79	242(24.97)	264(28.39)	506(26.65)
80–89	116(11.97)	113(12.15)	229(12.12)
90 +	17(1.8)	36(3.9)	53(2.8)
Total	969 (51.03)	930 (48.97)	1899
**Marital status**			
Married	279(28.79)	569(61.18)	848(44.66)
Not married	690(71.21)	361(38.82)	1051(55.34)
Total	969	930	2899
**Formal education**			
No	654(67.49)	473(50.86)	1127(59.35)
Yes	315(32.51)	457(49.14)	772(40.65)
Total	969	930	1899
**Education level**			
No	654(67.49)	473(50.81)	1,127(59.35)
Low	158(50.32)	204(44.54)	362(46.89)
Middle	138(43.95)	219(47.82)	357(46.24)
High	18(5.73)	35(7.64)	53(6.87)
Total	969	930	1899
**Residence**			
Rural	502(51.8)	451(48.5)	953(50.2)
Urban	467(48.2)	479(51.5)	946(49.8)
Total	969	930	1899
**Currently working**			
No	411(42.41)	311(33.44)	722(38.02)
Yes	558(57.59)	619(66.56)	1,177(61.98)
Total	969	630	1899
**Type of job**			
No	411(42.41)	311(33.44)	722(38.02)
Business	40(7.17)	34(5.49)	74(6.29)
Casual worker	7(1.25)	2(0.32)	9(0.76)
Employed	1(0.18)	2(0.32)	3(0.25)
Farming	510(91.40)	581(93.86)	1091(92.69)
Total	969	930	1899
**Financial provider in your family**			
Myself	531(54.80)	647(69.57)	1178(62.03)
Husband/wife	99(10.22)	39(4.19)	138(7.27)
Children	303(31.27)	216(23.23)	519(27.33)
Relatives/others	36(3.72)	28(3.01)	64(3.37)
Total	969	930	1899
**Type of cooking fuel**			
Electricity	2(0.21)	7(0.75)	9(0.47)
LPG (Gas)	9(0.93)	11(1.18)	20(1.05)
Kerosene	19(1.96)	6(0.65)	20(1.05)
Charcoal	171(17.65)	170(18.28)	341(17.96)
Firewood	721(74.41)	696(74.84)	1417(74.62)
Dung/others	47(4.85)	40(4.30)	87(4.59)
Total	969	930	1899
**Source of drinking water**			
Piped water around the house	61(6.29)	86(9.25)	147(7.74)
Public tap	258(26.63)	222(23.87)	480(25.28)
Water from well or borehole	648(66.88)	610(65.59)	1258(66.24)
Others	2(0.20)	12(1.29)	14(0.74)
Total	969	930	1899
**Socio-economic status**			
q1	325 (34)	223 (24)	548 (29)
q2	137 (14)	106(11)	243(13)
q3	193 (20)	158 (16)	351(18)
q4	163 (17)	218 (23)	381 (20)
q5	151 (16)	225 (24)	376 (20)
Total	969	930	1899(100)

Table 2 shows that 43.71% of the outpatient respondents paid cash directly for the service, and most of them paid themselves (47.99%). Among 744 respondents who attended the inpatient department, 64.78% paid cash directly for the services provided. Most of the respondents attended public health facilities (74.73%), while the rest attended private and charity or non-government organizations health facilities. Some respondents did not attend health facilities due to different reasons, including financial problems (37.95%).

**Table 2:** Summary of the respondents attended at a health facility: frequency (percentages) (*N* = 1,899)

Variables	Female	Male	Total
Did you pay for outpatient?			
Yes	412(42.52)	418(44.95)	830(43.71)
No	522(53.87)	482(51.83)	1004(52.87)
Don´t know	35(3.61)	30(3.23)	65(3.42)
Who paid for outpatient?			
Myself	165(38.92)	240(54.14)	405(47.99)
My wife/husband	27(6.37)	11(2.62)	38(4.50)
My child/children	178(41.98)	129(30.71)	307(36.37)
My relatives/others	54(12.74)	40(9.53)	94(11.14)
Did you pay for inpatient?			
Yes	246(62.44)	236(67.43)	482(64.78)
No	148(37.56)	114(32.57)	262(35.22)
Who paid for inpatient?			
Myself	65(21.24)	91(30.33)	156(25.74)
Wife/husband	7(2.29)	8(2.67)	15(2.48)
Children	146(47.71)	132(44)	278(45.87)
Non-family members	31(10.13)	26(8.67)	57(9.41)
Insurance	28(9.15)	26(8.67)	54(8.91)
Service was free	29(9.48)	17(5.67)	46(7.59)
In the last 12 months, have you ever stayed overnight in a hospital? (bed day)			
No	584 (60.27)	571 (64.40)	1155 (60.82)
Yes	385 (39.73)	359 (38.60)	744 (39.18)
In the last 12 months, how many different times were you a patient in a hospital for at least one night?			
Once	24 (62.60)	241 (67.13)	482 (64.78)
Twice	91 (23.64)	76 (21.17)	167 (22.45)
Three times	39 (10.13)	18 (5.01)	57 (7.66)
More than three times	14 (3.64)	24 (6.69)	38 (5.11)
What type of hospital or facility was it?			
Public	289 (75.06)	267 (74.37)	556 (74.73)
Private	64 (16.62)	67 (18.66)	131 (17.61)
Charity/NGOs	31 (8.05)	22 (6.13)	53 (7.12)
I don’t know	1 (0.26)	3 (0.84)	4 (0.54)
Have you (last 12 months) believed yourself to need hospitalisation but refrained from seeking care?			
Yes	409 (42.21)	372 (40.00)	781 (41.13)
No	560 (57.79)	558 (60.00)	1,119 (58.87)
What was/were the reason(s) why you did not seek medical care (more than one answer)?			
Problem cleared up	118 (27.00)	100 (24.94)	218 (26.01)
Financial problems	174 (39.82)	144 (35.95)	318 (37.95)
Other reasons	145 (33.18)	157 (39.15)	302 (36.04)

Table 3 indicates the relationship between the mean cost for both outpatient and inpatient care and EQ-5D scores. Results indicate that as EQ-5D scores increase, the cost for both inpatient and outpatient care decreases. This means that people with low QoL have higher costs for both outpatient and inpatient care compared to those with high QoL.

**Table 3:** Summary of average expenditures on access to outpatient and inpatient healthcare use and out-of-pocket (OOP) payment (*N* = 1,809)

EQ-5D Index	Number of people	Mean cost outpatient (US$)	Number of people	Mean cost inpatient (US$)	Mean OOP inpatient (US$)	Mean OOP outpatient (US$)
< = 0.109	10	14.08	8	37.9	9.47	11.68
0.110–0.209	6	65.53	6	83.71	10.76	6.19
0.210–0.309	18	13.32	14	75.63	10.27	6.38
0.310–0.409	14	13.09	12	23.3	2.85	6.30
0.410–0.509	27	9.29	17	40.78	3.30	7.33
0.510–0.609	285	10.46	208	36.03	4.97	6.56
0.610–0.709	131	8.68	94	30.29	3.80	6.18
0.710–0.809	245	9.82	173	32.61	3.72	6.20
0.810–0.909	107	10.49	69	34.65	2.21	6.44
0.910–1.00	222	7.92	143	40.3	4.04	6.93
Total	1065		744			

Key: Mean OOP = mean of out-of-pocket payment (lab test, medicines, and registration cost); Mean cost = mean of healthcare use (lab test, medicines, registration fees, cost per visit, bed day cost, and transport cost). The missing value in the number of patients affected the sample size which is classified as Missing Completely at Random (MCAR)

Table 4 indicates that the elderly have almost the same healthcare cost regardless of their social position (quintiles).

**Table 4:** The relationship between SES and mean cost for both outpatient and inpatient care (*N* = 1809)

**Socio-economic status**	**Number of people**	**Mean cost of outpatient care (US$)**	**Number of people**	**Mean cost of inpatient care (US$)**
q1	299	9.91	213	39.21
q2	152	7.90	111	26.55
q3	194	7.89	136	35.23
q4	203	10.71	140	43.76
q5	217	10.93	144	33.03
	**1065**		**744**	

Key: The missing value in the number of patients, affected the sample size which is classified as MCAR

In addition, there is a low mean for the OOP payments compared to the cost for the healthcare use for both outpatient and inpatient care.

### Regression analysis

Three simple linear regression analyses were performed to assess the factors associated with HE, which represent the cost of accessing outpatient and inpatient healthcare among the elderly.

Table 5 presents three simple linear regression analyses. Models 1 and 2 represent the relationship between HE for outpatient and inpatient care, respectively, with the independent variables of QoL, SES, and age. In these three linear regression analyses, we found an association between HE for both outpatient and inpatient care and QoL, which is statistically significant. This means that HE for outpatient and inpatient care increases with the decrease of QoL. For age, although it seems only one group was statistically significant in all three linear regression analyses, it indicates that as age increases, the HE for outpatient and inpatient care increases. All quintiles for the SES of the elderly were not statistically significant to explain the HE for outpatient and inpatient care. This means that SES of the elderly had no influence on the HE for outpatient and inpatient care since the mean cost for inpatient care had almost the same values in all groups.

**Table 5:** Linear regression analysis for both outpatient and inpatient care

Model 1					Model 2			
HE (OP) = QoL + Age					**HE (IP) = QoL + Age**			
	Coef	P >|t|	95% CI		Coef	P >|t|	95% CI	
QoL	-9.406	0.000	-14.551	-4.262	-27.280	0.005	-46.357	-8.203
Age								
60–69	0							
70–79	-0.801	0.510	-3.184	1.582	1.533	0.720	-6.857	9.922
80–89	3.187	0.045	0.071	6.303	13.803	0.017	2.425	25.181
90 +	4.398	0.159	-1.719	10.514	-9.196	0.416	-31.343	12.951
HE (OP) = SES + Age					**HE (IP) = SES + Age**			
SES								
q1	0							
q2	-2.087	0.209	-5.346	1.173	9.140	0.137	-2.912	21.192
q3	-0.287	0.852	-3.300	2.726	-4.557	0.404	-15.264	6.150
q4	0.853	573	-2.117	3.823	-3.838	0.471	-14.267	6.591
q5	1.025	0.490	-1.890	3.940	-3.693	0.489	-14.166	6.780
Age								
60–69	0							
70–79	-0.456	0.709	-2.851	1.939	2.146	0.616	-6.244	10.536
80–89	3.911	0.014	0.796	7.027	15.287	0.008	3.922	26.653
90 +	6.097	0.051	-0.015	12.2101	-5.264	0.639	-27.290	16.762
HE (OP) = QoL + SES + Age					**HE (IP) = QoL + SES + Age**			
QoL	-9.786	0.000	-14.949	-4.623	-26. 920	0.006	-46.041	-7.800
SES								
q1	0							
q2	-2.333	0.158	-5.576	0.909	8.750	0.154	-3.284	20.784
q3	-0.631	0.680	-3.632	2.369	-5.170	0.343	-15.867	5.527
q4	0.782	0.604	-2.171	3.734	-3.575	0.501	-13.987	6.838
q5	1.166	0.430	-1.732	4.065	-3.279	0.539	-13.737	7.179
Age								
60–69	0							
70–79	-0.715	0. 556	-3.100	1. 669	1.366	0.750	-7. 028	9.759
80–89	3.233	0.042	0. 116	6.350	13. 820	0.017	2. 426	25. 213
90 +	4.634	0.138	-1.490	10.759	-9. 365	0.408	-31. 544	12.815

Key: *OP* outpatient care, *IP* inpatient care

## Discussion

This study analysed the association between measures of health status (QoL), health cost (HE), and social position (SES) among the elderly in Igunga and Nzega districts. The main findings of this study indicate an association between poor health of the elderly (low QoL) and high costs for healthcare and no association between social position (SES) and health costs for healthcare among the elderly. This means that the elderly with poorer health use more healthcare compared to those with good health, regardless of their social position. Similar findings were reported in India and elsewhere [33–35] that the elderly with chronic diseases and poor health reduced their health status, which, in return, leads to an increase of HE.

As was expected, the study found an association between age and HE. This is because as age increases, the health status of the elderly starts to deteriorate, with a decrease of QoL, which, in return, increases the HE among the elderly to improve their health status. The findings concur with previous studies done in Tanzania and elsewhere [17, 20, 23, 36, 37], which revealed that as age increases, the health status of the elderly decreases and the HE increases.

In LMICs, most of the elderly suffer from catastrophic HEs due to not being well defined in the financing healthcare service system and other policies, including the exemption policy for the elderly. However, the study found that health spending does not depend on the level of SES of the individual. These findings concur with other studies done in China, Burkina Faso, and some countries in Asia [38–40]. In addition, the findings differ from some studies done in the United States of America and elsewhere where it was found that there is a positive relationship between SES and access to health care services and health spending [41]. On the other hand, studies done in the Netherlands found that households with low incomes had higher health expenditures compared with households with higher incomes [42, 43]. The influence of social position could be clearer in the long term than in the short term. In the short term, when the elderly are ill, they need treatment, and after receiving the treatment they can improve their health status whether they are poor or rich. In the long term, both QoL and HE are influenced by the level of social position (SES) [43–45].

### Policy implications

There have been many efforts to improve healthcare services to all Tanzanians, which are well established in different documents, including the Tanzania Development Vision 2025 (TDV 2025), the National Health Policy 2017, the National Five Year Development Plan 2016/17 – 2020/21, the Sustainable Development Goals 2030, and the Health Sector Strategic Plan 2015 – 2020 [46]. However, there are still many challenges facing the elderly in accessing free or almost free healthcare services. Due to these challenges, local government authorities (LGAs) are obliged to handle the priority setting process in a way that can benefit vulnerable groups like the elderly. The decentralised health system at LGAs should design policies to protect the elderly with lower SES. This can be done by LGAs using the bottom-up decision-making system, whereby the priority setting process starts from the lower level by reorganising the problems around individuals and proposes good measures. The government, especially LGAs, when setting their priorities under a decentralised health system should consider the interdependence of health, wealth, and HE to improve the health among the elderly. This can be done by increasing the budget for the elderly through subsidising the healthcare services of the elderly. To help the elderly, the government can also establish health insurance (risk pooling) specifically for vulnerable groups, including the elderly. This will help to reduce the challenges of exemption policy in accessing health services among the elderly.

### Strengths and limitations

The strength of this study lies in the fact that it provides information on the association among health (QoL), social position (SES), HE, and age among the elderly, which may contribute to the priority setting during planning for the resource allocation to the vulnerable groups, including the elderly especially at the LGAs in rural Tanzania. The study used cross-sectional data, which may not capture the causality of the problems facing the elderly in financing the healthcare services to reduce the burden of diseases. This may be one of the limitations of the study. Another limitation is self-reported data that might have created recall bias and heterogeneity among the elderly on socio-economic factors (monthly income/wealth) and self-rated health data. Recall bias might influence the validity and reliability of the study findings. These problems were minimised by conducting a pilot test of the survey tool, which helped to refine some of the questions. During interviews, questions were elaborated to the respondents.

## Conclusion

The health system in these districts allocates resources mainly according to needs, and social position does not influence them. A relatively small proportion of the total costs for healthcare is financed by OOP payments. We thus conclude that the system is reasonably fair. Health, not wealth, determines the use of medical expenditures.

## Data Availability

datasets used and/or analyzed during the current study available from the corresponding author on reasonable request.

## Abbreviations

CI: Confidence Interval
EQ-5D: European Quality of Life Five Dimension
FBO: Faith-Based Organisation
CHFs: Community Health Funds
GDP: Gross Domestic Product
HE: Health Expenditure
KMO: Kaiser–Meyer–Olkin
LGAs: Local Government Authorities
LMICs: Low- and Middle-Income Countries
MCAR: Missing Completely at Random
OOP: Out-of-Pocket
PCA: Principal Component Analysis
QALYs: Quality-Adjusted Life Years
QoL: Quality of Life
SAGE: Study on global AGEing and adult health
SES: Socio-economic Position
WHO: World Health Organization

## Footnotes

1 WHO, https://www.who.int/choice/country/country_specific/en/ accessed on Friday, 11^th^ October 2019, 9:08 am.
